# Post-ERCP bacteremia caused by *Alcaligenes xylosoxidans *in a patient with pancreas cancer

**DOI:** 10.1186/1476-0711-5-19

**Published:** 2006-09-01

**Authors:** Gurdal Yilmaz, Kemalettin Aydin, Iftihar Koksal, Rahmet Caylan, Korhan Akcay, Mehmet Arslan

**Affiliations:** 1 Department of Infectious Diseases and Clinical Microbiology, Karadeniz Technical University School of Medicine, Trabzon, Turkey; 2 Department of Gastroenterology, Karadeniz Technical University School of Medicine, Trabzon, Turkey

## Abstract

*Alcaligenes xylosoxidans *is an aerobic, motile, oxidase and catalase positive, nonfermentative Gram negative bacillus. This bacterium has been isolated from intestine of humans and from various hospital or environmental water sources. *A.xylosoxidans *is both waterborne and results from the poor-hygienic conditions healthcare workers are in. In this case report, the bacteremia which appeared in a patient with pancreas cancer after ERCP was described.

## Background

Bacteremia is a rare complication of endoscopic retrograde cholangiopancreatography (ERCP) and biliary stents. The rate of post-ERCP cholangitis and sepsis ranges from 0.5% to 3.0% [1,2].

*Alcaligenes xylosoxidans *is a rare cause of bacteremia. This organism, also known as *Achromobacter xylosoxidans*, is an aerobic, motile, oxidase and catalase positive, nonfermentative Gram negative bacillus. *A.xylosoxidans *is opportunistic and usually affects severely immunocompromised patients such as those with neutropenia and those with a malignant or cardiovascular disease [3,4]. This microorganism has been isolated from blood, cerebrospinal fluid, stool, urine, sputum, peritoneal fluid, skin, ear discharge, wounds, abscesses, bone, joints, endocardium and central venous catheters [3-8].

In the present report is described a case with bacteremia due to *A.xylosoxidans *post-ERCP in patient of pancreas cancer.

## Case report

A 70-year-old man was admitted to our hospital with a 10-day history of jaundice and abdominal pain. The patient is known to have suffered from pancreas cancer for three months and he was received second cycle of chemotherapy before one month. His vitality signs were: blood pressure was 110/70 mmHg, body temperature 36.3°C and pulse rate 68/min. His peripheral white blood cell count was 6.4 × 10^9^/L, erythrocyte sedimentation rate was 72 mm/h and C-reactive protein was 4.6 mg/dL. Four days later, the stent was placed into the biliary tract with ERCP. One day later, the patient was lethargic. His vitality signs were: blood pressure was 90/50 mmHg, body temperature 39.7°C and pulse rate 112/min. His peripheral white blood cell count was 14.1 × 10^9^/L with 86% neutrophils and 8% lymphocytes. His erythrocyte sedimentation rate was 80 mm/h and C-reactive protein was 11.2 mg/dL. Blood and urine specimens were taken for microbiologycal analysis. We started to administer empirical treatment with ceftriaxone (1000 mg per 12 h; IV) to the patient. In blood culture (Bactec 9240; Becton Dickinson, Sparks, Md.), Gram negative bacillus was found to have reproduced. This microorganism identified with the help of Phoenix system (Becton Dickinson, Sparks, Md.) and biochemical tests. It was called as *A.xylosoxidans*. *A.xylosoxidans *was distinguished from other Alcaligenes species by acidification of oxidative-fermentative (OF) glucose and xylose. Key characteristics of *A.xylosoxidans *are shown in Table 1.

**Table 1:** Key characteristics of *A.xylosoxidans*

**Tests**	**Results**
Oxidase	+
Catalase	+
OF xylose	Acid reaction
OF glucose	Acid reaction
Arginine	-
Citrate	+
Ketoglutaric acid	+
Gamma glutamil	+
NO_3 _to NO_2_	+
Acetamide	+
Lysine	-
Mannitol	-
Urease	-
Motility	+

The urine culture was sterile. Three days later, the initial treatment was modified to ciprofloxacine (200 mg per 12 h; IV) according to antimicrobial susceptibility test. In-vitro susceptibility data are shown in Table 2. This isolate is an ESBL producer. Five days later, the clinical condition of the patient improved. He was discharged in a good clinical condition after 15 days.

**Table 2:** In-vitro susceptibility profile of *A.xylosoxidans*

**Antimicrobial agent**	**Susceptibility**
Amikacin	Resistant
Cefoperazone/sulbactam	Sensitive
Cefotaxime	Resistant
Ceftazidime	Resistant
Ceftriaxone	Resistant
Ciprofloxacin	Sensitive
Imipenem	Sensitive
Piperacillin/tazobactam	Sensitive
Tobramycin	Resistant
Trimethoprim/sulfametoxazole	Sensitive

## Discussion

Obstruction of the bile duct by stones or tumor can facilitate bacterial colonization; subsequent instrumentation has resulted in bacteremia rates mean 18.0%. [9,10]. The highest bacteremia rates are seen in therapeutic ERCP. In purely diagnostic ERCP, the bacteremic rate is lower at 8% [10,11]. The microorganism most responsible for post-ERCP bacteremia is *Escherichia coli *[9] . *A.xylosoxidans *is a rare but important cause of bacteremia in immunocompromised patients. The gastrointestinal tract has been suggested as a source for *A.xylosoxidans *bacteremia in patients with cancer [12]. Our case report is the first one associated with *A.xylosoxidans *that causes post-ERCP bacteremia.

*A.xylosoxidans *has been isolated from intestine of humans and from various hospital or environmental water sources [13]. The natural sources of *A.xylosoxidans *infections are well water, tap water, swimming pools, and moist soil [14,15]. *A.xylosoxidans *causing nosocomial infections is waterborne (disinfectant solutions, intravenous fluids, dialysis solutions) and results from the fact that healthcare workers do not use gloves [13,15,16]. In our case, peripheral factors wereanalysed as a source of infection but any environmental contamination couldn't be indicated. Thatthe patient had symptoms of infection one day after ERCP made us think that the infection was from the intestines.

*A.xylosoxidans *is a weakly virulent microorganism. In general, there is an underlying dissease in patients. *A.xylosoxidans *have been reported in patients with cancer, neutropenia, bone marrow or liver transplant, renal failure, cystic fibrosis, HIV infection, IgM deficiency, neonates [4-6,15,17] .

This report showed that *A.xylosoxidans *was sensitive to cefoperazone/sulbactam, ciprofloxacin, imipenem, piperacillin/tazobactam and trimethoprim/sulfametoxazole and resistant to the third generation cephalosporins with the exception of the cefoperazone/sulbactam, amikacin and tobramycin. In previous studies, it was reported that *A.xylosoxidans *was resistant to most of the antimicrobial agents [15,17,18].

In summary, the post-ERCP bacteremia caused by *A.xylosoxidans *was presented in a 70-year-old man with pancreas cancer. The case report may help to redefine the role of *A.xylosoxidans *in post ERCP infections. The association of *A.xylosoxidans *with bacteremia further extends the clinical spectrum of this rare pathogen. This unusual case highlights that an effective antimicrobial therapy based on an immediate microbiologycal analysis may be life-saving in patients presenting a severe complication of ERCP.
